# Differences between Self-Report and Biomarkers in Smoking Indicators: The Necessity of Biomonitoring in Global Surveillance

**DOI:** 10.3390/ijerph20031801

**Published:** 2023-01-18

**Authors:** Myung-Bae Park

**Affiliations:** Department of Health and Welfare, Paichai University, 155-40 Baejae-ro, Seo-gu, Daejeon 3345, Republic of Korea; parklove5004@naver.com or parkmb@pcu.ac.kr; Tel.: +82425205037

## 1. Introduction

Tobacco causes premature death through cardiovascular disease, cancer, and respiratory disease. It has adverse effects on all areas of health, including mental health, with an increased risk of suicide and depression [1,2,3,4]. There are more than 1.3 billion smokers worldwide, and more than half of them will die from smoking-related diseases [5]. Smoking is linked to greater mortality than the combined effects of drinking alcohol, traffic accidents, and acquired immune deficiency syndrome. It is estimated that smoking causes nearly 9 million deaths each year, of which 1.2 million are due to second-hand smoke [3,5]. As of 2020, smokers are estimated to comprise 22.3% of the global adult population, with a higher prevalence in males, accounting for 36.7% of that cohort compared to 7.8% of females [5].

Tobacco is a clear, leading preventable cause of death. Reducing tobacco consumption and exposure has become a significant public health priority. The World Health Organization (WHO) enacted the Framework Convention on Tobacco Control (FCTC), the first international treaty in the field of health, in 2003 and is working and cooperating worldwide for tobacco control [6]. Furthermore, the Centers for Disease Control and Prevention (CDC) in the U.S. manages tobacco use as a key risk factor in preventing the four major chronic diseases: cardiovascular diseases, chronic respiratory diseases, cancers, and diabetes [7].

## 2. Measurement of the Prevalence of Active and Passive Smoking

The most important factor in policy making, including tobacco control policies, is to investigate the current status based on accurate statistics. It is crucial to determine how tobacco-related indices, such as smoking or exposure prevalence, are generated and whether they are accurate. There have been consistent study findings that, although most countries utilize self-reported (SR) data for their official statistics, this does not accurately reflect reality [8].

There are several methods for measuring smoking-related indices, and the most common are SR and biomarkers. SR is the most widely used method to survey populations, owing to its convenience and economic advantage and the ability to examine the indices in various situations and environments. However, tobacco use tends to be underestimated due to social desirability response bias [9,10,11]. In Confucian-influenced Asian countries, including South Korea, false responses are more common due to social desirability. Tobacco use has been reported to be more than twofold higher than the official statistics suggest among females [11]. Adolescents are another group heavily influenced by social desirability. For this reason, smoking-related indices among adolescents are sometimes investigated using online instead of face-to-face surveys [9,12]. However, even an online survey cannot eliminate errors linked to the method of investigation, and the risk of bias (risk for underestimation) remains high [12].

One investigation method that is not affected by such bias is the use of biomarkers. Biomarkers are the most accurate indices for tobacco use and smoking exposure [13]. Nicotine and cotinine are the most widely used substances. Cotinine is more often used in population surveys owing to its relatively more appropriate half-life and convenience of sampling. Detection of cotinine in a biological sample at a certain threshold or higher indicates current smoking or exposure to passive smoking [14].

Biomarkers overcome the subjectivity and inaccuracy of SR, can be used to identify current smokers, and reflect exposure to second-hand smoke in all places. SR-related bias can identify current smokers and individuals exposed to second-hand smoke [15,16]. Most existing second-hand smoke surveys ask about possible recent (e.g., in the past seven days) exposure to tobacco smoke, such as “Did someone else smoke cigarettes or other tobacco products indoors?” [17]. However, this method is vulnerable to a few measurement errors. The questions ask about exposure to other people’s smoking at home, work, or other public places, ignoring that individuals can be exposed to second-hand smoke in other areas. Although some questions ask about other places, accurate responses are often hindered due to inaccurate recall and an inability to remember all exposure situations in the past. In other words, the respondent’s memory limitations and unrecognized exposure cannot be measured. Moreover, interest in thirdhand smoke (THS) has grown in recent years. THS refers to exposure to indirect smoking through tobacco residue that is adsorbed into surfaces within an environment and is re-emitted to the individual remaining in the same space afterward [18,19]. Studies have reported that individuals in these circumstances do not recognize tobacco smoke, and even short-term exposure through THS can elevate cotinine concentrations in the body [20]. SR only reflects second-hand smoke and cannot reflect THS. In other words, SR cannot fully reflect smoking exposure.

Therefore, it is not necessarily the case that biomarkers are the best method. Biomarkers are, for instance, more costly than SR; thus, it is not easy to use screening for large-scale surveys. Furthermore, biomarker concentration only shows current smoking or indirect smoking exposure and severity, and cannot shed light on the details of context and situation, such as smoking habits, places, and reasons.

## 3. The Necessity of Biomonitoring in Global Surveillance: Lessons from the US, UK, and South Korea

Few developed countries, including the United States, United Kingdom, Canada, Korea, and Poland, measure biomarkers in national health surveys [21,22,23,24]. Therefore, biomarkers cannot be established as the standard international indices. Nevertheless, biomarkers should be recommended as smoking-related indices reported by international organizations, such as the World Health Organization (WHO), the Organisation for Economic Cooperation and Development (OECD), and the European Union (EU). The generation of accurate statistical data is critical to policy making. Many studies have utilized biomonitoring and reported that the actual smoking population would be larger than the officially reported statistics [9,10,11,12,18]. Thus, biomarkers must be utilized as supplements to reflect reality better.

Regarding passive smoking, the United States has already set the reduction of serum cotinine concentration, as opposed to the decrease in smoking on SR, as the target in Healthy People 2030 [21,25]. The UK monitors the passive smoking rate based on SR and saliva cotinine concentrations in their annual Health Survey for England. Unfortunately, no other countries around the world utilize biomarkers in official statistics or as part of the policy agenda despite existing study findings on the usefulness of biomarkers. In Korea, urine cotinine measurement started in 2008. However, it has never been published as an official statistic or an agenda of the Health Plan. Many studies conducted in Korea have already reported that the current smoking rate, determined based on cotinine concentration markedly, differs from that calculated based on SR. Furthermore, the official passive smoking exposure rate based on SR has substantially declined in the past ten years. Furthermore, studies have shown that the exposure rate does not decrease when ‘no exposure’ is defined as a cotinine level below the limit of detection (LOD), a method used by the US, but biomarkers are still not used. Therefore, Asian countries must implement biomonitoring in national health policies. Recommendations by international organizations, such as the WHO and OECD, can facilitate countries currently measuring biomarkers in their national health surveys to implement biomonitoring officially. In addition, several studies have compared SR and biomarkers in specific population subsets in various countries. Establishing such a reporting system will promote the use of biomarkers as supplementary data to determine current tobacco-related status and evidence for policy making, even in countries that do not investigate biomarkers as part of their national health surveys.

## Data Availability

Not applicable.

## References

[1] Drope J., Schluger N.W. (2018). The Tobacco Atlas.

[2] Pitchot W., Hansenne M., Ansseau M. (2001). Role of dopamine in non-depressed patients with a history of suicide attempts. Eur. Psychiatry.

[3] CDC (2021). Health Effects of Cigarette Smoking. https://www.cdc.gov/tobacco/data_statistics/fact_sheets/health_effects/effects_cig_smoking/index.htm.

[4] Park M.-B., Kwan Y., Sim B., Lee J. (2021). Association between urine cotinine and depressive symptoms in non-smokers: National representative sample in Korea. J. Affect. Disord..

[5] WHO (2022). Tobacco. https://www.who.int/news-room/fact-sheets/detail/tobacco.

[6] WHO (2003). WHO Framework Convention on Tobacco Control.

[7] CDC (2022). Tobacco Use.

[8] West R., Zatonski W., Przewozniak K., Jarvis M.J. (2007). Can we trust national smoking prevalence figures? Discrepancies between biochemically assessed and self-reported smoking rates in three countries. Cancer Epidemiol. Biomark. Prev..

[9] Klein J.D., Thomas R.K., Sutter E.J. (2007). Self-reported smoking in online surveys prevalence estimate validity and item format effects. Med. Care.

[10] Gorber S.C., Schofield-Hurwitz S., Hardt J., Levasseur G., Tremblay M. (2009). The accuracy of self-reported smoking: A systematic review of the relationship between self-reported and cotinine-assessed smoking status. Nicotine Tob. Res..

[11] Park M.B., Kim C.-B., Nam E.W., Hong K.S. (2014). Does South Korea have hidden female smokers: Discrepancies in smoking rates between self-reports and urinary cotinine level. BMC Women’s Health.

[12] Park M.B., Nam E.W., Lee S.K., Kim C.B., Ranabhat C. (2015). The correlation of different cotinine levels with questionnaire results: A comparative study for different measurement methods of the adolescent smoking rate in Korea. Asia Pac. J. Public Health.

[13] Benowitz N.L. (1996). Cotinine as a biomarker of environmental tobacco smoke exposure. Epidemiol. Rev..

[14] Avila-Tang E., Al-Delaimy W.K., Ashley D.L., Benowitz N., Bernert J.T., Kim S., Samet J.M., Hecht S.S. (2013). Assessing second-hand smoke using biological markers. Tob. Control.

[15] Apelberg B.J., Hepp L.M., Avila-Tang E., Gundel L., Hammond S.K., Hovell M.F., Hyland A., Klepeis N.E., Madsen C.C., Navas-Acien A. (2013). Environmental monitoring of second-hand smoke exposure. Tob. Control.

[16] Hsieh S.J., Ware L.B., Eisner M.D., Yu L., Jacob P., Havel C., Goniewicz M.L., Matthay M.A., Benowitz N.L., Calfee C.S. (2011). Biomarkers increase detection of active smoking and second-hand smoke exposure in critically ill patients. Crit. Care Med..

[17] Avila-Tang E., Elf J.L., Cummings K.M., Fong G.T., Hovell M.F., Klein J.D., McMillen R., Winickoff J.P., Samet J.M. (2013). Assessing second-hand smoke exposure with reported measures. Tob. Control.

[18] Sim B., Park M.-B. (2021). Exposure to Secondhand Smoke: Inconsistency between Self-Response and Urine Cotinine Biomarker Based on Korean National Data during 2009–2018. Int. J. Environ. Res. Public Health.

[19] Park M.-B., Lee T.S., Oh J.E., Lee D.H. (2022). Thirdhand smoke exposure: Differences in smoke exposure indices and cultural norms between hotels and motels in South Korea. Indoor Built Environ..

[20] Park M.-B., Sim B. (2022). Evaluation of Thirdhand Smoke Exposure after Short Visits to Public Facilities (Noraebang and Internet Cafés): A Prospective Cohort Study. Toxics.

[21] NIH (2019). Second-hand Smoke Exposure.

[22] Wong S.L., Malaison E., Hammond D., Leatherdale S.T. (2013). Second-hand smoke exposure among Canadians: Cotinine and self-report measures from the Canadian Health Measures Survey 2007–2009. Nicotine Tob. Res..

[23] Jarvis M.J., Feyerabend C. (2015). Recent trends in children’s exposure to second-hand smoke in England: Cotinine evidence from the Health Survey for England. Addiction.

[24] CDC (2015). Second-Hand Smoke: An Unequal Danger.

[25] OASH (2022). Reduce the Proportion of People Who Don’t Smoke but Are Exposed to Second-Hand Smoke.

